# Isolation and characterization of olfactory ecto-mesenchymal stem cells from eight mammalian genera

**DOI:** 10.1186/s12917-018-1342-2

**Published:** 2018-01-17

**Authors:** Antoine D. Veron, Cécile Bienboire-Frosini, François Feron, Elisa Codecasa, Arnaud Deveze, Dany Royer, Paul Watelet, Pietro Asproni, Kevin Sadelli, Camille Chabaud, Jean-claude Stamegna, Joël Fagot, Michel Khrestchatisky, Alessandro Cozzi, François S. Roman, Patrick Pageat, Manuel Mengoli, Stéphane D. Girard

**Affiliations:** 1 IRSEA, Research Institute in Semiochemistry and Applied Ethology, Quartier Salignan, 84400 Apt, France; 2 Aix Marseille Univ, CNRS, NICN, Marseille, France; 3 Inserm CBT 1409, Centre d’Investigations Cliniques en Biothérapie, Marseille, France; 4 Département ORL, Hôpital Universitaire Nord, AP-HM, Marseille, France; 5 Aix-Marseille Univ, IFSTTAR, LBA, Marseille, France; 6 Centre Hospitalier Vétérinaire Pommery, 51100 Reims, France; 7 Société Hippique Le frigouyé, 30650 Saze, France; 8 Aix-Marseille Univ, CNRS, LPC, Marseille, France; 9 Present address: Vect-Horus S.A.S., Faculté de Médecine Secteur Nord, CS80011, Boulevard Pierre Dramard, 13344 Marseille, Cedex 15 France

**Keywords:** Adult craniofacial stem cells, Dog, Ecto-mesenchymal stem cells, Horse, Non-human primate, Rabbit, Regenerative medicine, Rodent, Sheep, Veterinary medicine

## Abstract

**Background:**

Stem cell-based therapies are an attractive option to promote regeneration and repair defective tissues and organs. Thanks to their multipotency, high proliferation rate and the lack of major ethical limitations, “olfactory ecto-mesenchymal stem cells” (OE-MSCs) have been described as a promising candidate to treat a variety of damaged tissues. Easily accessible in the nasal cavity of most mammals, these cells are highly suitable for autologous cell-based therapies and do not face issues associated with other stem cells. However, their clinical use in humans and animals is limited due to a lack of preclinical studies on autologous transplantation and because no well-established methods currently exist to cultivate these cells. Here we evaluated the feasibility of collecting, purifying and amplifying OE-MSCs from different mammalian genera with the goal of promoting their interest in veterinary regenerative medicine.

Biopsies of olfactory mucosa from eight mammalian genera (mouse, rat, rabbit, sheep, dog, horse, gray mouse lemur and macaque) were collected, using techniques derived from those previously used in humans and rats. The possibility of amplifying these cells and their stemness features and differentiation capability were then evaluated.

**Results:**

Biopsies were successfully performed on olfactory mucosa without requiring the sacrifice of the donor animal, except mice. Cell populations were rapidly generated from olfactory mucosa explants. These cells displayed similar key features of their human counterparts: a fibroblastic morphology, a robust expression of nestin, an ability to form spheres and similar expression of surface markers (CD44, CD73). Moreover, most of them also exhibited high proliferation rates and clonogenicity with genus-specific properties. Finally, OE-MSCs also showed the ability to differentiate into mesodermal lineages.

**Conclusions:**

This article describes for the first time how millions of OE-MSCs can be quickly and easily obtained from different mammalian genera through protocols that are well-suited for autologous transplantations. Moreover, their multipotency makes them relevant to evaluate therapeutic application in a wide variety of tissue injury models. This study paves the way for the development of new fundamental and clinical studies based on OE-MSCs transplantation and suggests their interest in veterinary medicine.

## Background

Stem cell-based regenerative medicine is an innovative field of scientific investigation that provides reliable evidence for repairing damaged tissues and organs, both in human and veterinary medicine [1–3]. Being plastic and self-renewing, stem cells have been proposed as a potential treatment for a variety of disorders [4–9]. However, the development of such therapies remains a scientific challenge. Currently, the use of stem cells or their derivatives in human medicine is still limited to a small number of applications: hematopoietic stem cell are the only type of grafted stem cell routinely used in clinics [10]. In veterinary medicine, stem cell-based regenerative therapies are a fast growing field of research, particularly with the development of new treatments for musculoskeletal injuries [1, 2]. However, the clinical use of stem cells in veterinary medicine is in its early stages and pre-clinical studies aimed in determining the most suitable stem cell types and modes of delivery are still required [11, 12].

Among the potential stem cell candidates for regenerative therapy, adult nasal stem cells present in the olfactory mucosa are a promising candidate both for human and veterinary medicine [13–16]. This easily accessible peripheral tissue contains highly proliferative stem cells that do not face ethical or technical issues associated with other stem cells types [17–20]. Characterized as a member of the mesenchymal stem cell (MSC) superfamily, these cells are known as “olfactory ecto-mesenchymal stem cells” (OE-MSCs) [14]. OE-MSCs are located in a permanently self-renewing nervous tissue with an ectodermal embryonic origin [21, 22] and assumed to be derived from neural crest cells, like other adult stem cells of the craniofacial area [23]. They are multipotent, providing a potential source of stem cells for treating numerous types of tissue damages [13, 14]. Moreover, human OE-MSCs can be quickly and easily propagated in sufficient numbers to meet the requirements for cell transplantation without showing tumorigenicity risks [14, 24–26]. Although the therapeutic potential of OE-MSCs has not yet been assessed in human or veterinary medicine, their therapeutic effect has been evaluated in various rodent models of tissue damage such as myocardial infarct [27], spinal cord trauma [28–30], cochlear damage [31], Parkinson’s disease [32], and ischemic/hypoxic injury of the hippocampus [25, 33]. Despite the promising results reported by these studies, their clinical use in humans and animals is limited by the lack of well-established methods for the collection of these cells from living animals, and for their purification and amplification. To overcome this problem, the authors of the present study recently developed an efficient and minimally invasive procedure for autologous transplantation of OE-MSCs in rats [34]. According to this method, each animal is the donor as well as the receiver of its own cells, thereby excluding complications and side effects associated with other grafting methods.

The present study evaluated the feasibility of collecting, purifying and amplifying OE-MSCs from living animals belonging to eight genera of mammals, relevant for basic research or clinical veterinary practice. In the prospect of future autologous transplantation therapies, we also assessed OE-MSCs stemness features.

## Methods

### Collection of olfactory mucosa from different mammalian genera

Biopsies of nasal olfactory mucosa were obtained as previously described in humans and rats with some modifications. According to the morphology and accessibility of the nasal cavity, three protocols were used to access the olfactory mucosa. In mice, muzzle was dissected as previously described in euthanized rats [20]. For rats and gray mouse lemurs, a less invasive method, requiring nasal bone perforation at the junction with the frontal bone, was used to access the olfactory mucosa, as previously described in living rats [34]. In all other genera, biopsies were directly obtained by nasal cavity exploration with pliers, as previously described in humans [20, 35]. Details for each genus and the strategies used for biopsies are summarized in Table 1. For all genera, the fragments of olfactory mucosa were placed, immediately after excision, in culture medium [Dulbecco’s Modified Eagle’s Medium/Ham’s F12 (DMEM/F12) supplemented with 10% Fetal Bovine Serum (FBS), 200 units/mL of penicillin, 200 μg/mL of streptomycin (Life Technologies), 0.25 μg/ml of Amphotericin B (Fungizone, Sigma/Aldrich) and 12.5 μg/ml of Plasmocin Treatment (InvioGen)]. For horse cells, the culture medium was supplemented with 1 μg/ml of Ketoconazole (Sigma/Aldrich) to prevent fungal contamination due to stabling. Then, the pieces of olfactory mucosa placed in the culture medium were stored in a refrigerated container until processing.

**Table 1:** Subject and procedures for collection of olfactory mucosa

Genus (*species*)	Gender & population	Strain & Origin	Age	Anaesthesia & analgesia procedure	Collection method
Mouse (*Mus musculus*)	Male, 6^a^	C57Bl/6NCrl, Charles River Laboratories	3 months	Euthanasia; sodium pentobarbital (Ceva Santé Animale, 200 mg/kg, i.p.)	Muzzle dissection
Rat (*Rattus norvegicus*)	Male, 6^a^	Fischer 344, Charles River Laboratories	6 weeks	Sodium pentobarbital (Nembutal, 60 mg/kg, i.p.) and Buprenorphine (0.02 mg/kg i.m.)	Nasal bone trepanation
Rabbit (*Oryctolagus cuniculus*)	Male, 3 Female,3	Breed**,** IRSEA	11 (± 1.5) months	Medetomidine (Domitor®, 0.4 mg/kg i.m.), Ketamine 100 (Imalgene®, 30 mg/kg i.m.) and Buprenorphine (0.02 mg/kg i.m.)	Nasal cavity exploration
Dog (*Canis lupus*)	Male, 4 Female, 2	Multiple, pet dogs^b^	15 (± 1) years	Zoletil (Tiletamine and Zolazepam,Virbac, 5 mg/kg i.v.) and Boutorfanole (Dolorex®, 0.1 mg/kg i.v.)	Nasal cavity exploration
Horse (*Equus caballus*)	Male, 4	Multiple, client-owned^b^	18 (± 3) years	Détomidine (Domosedan®, 10–20 μg/kg i.v.)	Nasal cavity exploration
Sheep (*Ovis aries*)	Female, 3	Merinos**,** IRSEA	7 (± 4) years	Xylazine (Rompun® 2%, 0.04 mg/kg i.v.)	Nasal cavity exploration
Gray mouse Lemur (*Microcebus murinus*)	Male, 1 Female, 1	CNRS Primatology centre	7 years	Diazepam (Valium, 20 mg/kg i.m.) and Ketamine (Imalgene, 100 mg/kg i.p.)	Nasal bone trepanation
Macaque (*Macaca fascicularis***)**	Male, 1	CNRS Primatology centre	13 years	Zoletil (Tiletamine and Zolazepam,Virbac, 5 mg/kg i.m.); then isoflurane (Belamont)	Nasal cavity exploration

^a^mucosa from different individuals were pooled

^b^client-owned animals from veterinary clinics biopsied with owner consent

Anesthesia and surgical procedures were performed according to the European law on Animal Care Guidelines and the Animal Care Committee of Aix-Marseille University and Ethic Committee of IRSEA (C2EA125) approved our protocols.

### Isolation and expansion of OE-MSCs

Cultures of OE-MSCs were carried out as previously described by the authors [34]. Following biopsy, pieces of olfactory mucosa were washed twice in DMEM/ F12 medium. The biopsies were then dissected into small pieces using 25-gauge needles (< 1 mm^2^). Each explant was plated on a 2 cm^2^ culture well coated with poly-L-lysine (5 μg/cm^2^ in sterile water, Sigma-Aldrich). Except for rodents, the mucosal pieces were covered with a sterile glass coverslip to facilitate and accelerate the adhesion of the explants at the bottom of the culture wells. The wells were filled with 250 μl of the culture medium described above. One week later, concentrations of antibiotics were reduced (100 units/mL of penicillin and to 100 μg/mL of streptomycin), Amphotericin B and Ketoconazole were removed and Plasmocin Treatment was replaced by 1.25 μg/ml Plasmocin Prophylactic (InvivoGen). This medium is referred as growth medium throughout the manuscript. Culture medium was gently renewed every 2 to 3 days. After an additional week, the cells began to grow out of the explants and invaded the culture dish. When confluency was reached, the cells were detached using a trypsin-EDTA solution (0.05%, Life Technologies), pooled and centrifuged at 300 x g for 5 min and replated without exceeding a 1:3 cell split ratio. When several individuals of a species were biopsied, cells from different cultures were compared.

### Generation of spheres

For generating spheres, cells were counted in duplicate using an automated counter (Scepter, Millipore) and plated at a density ranging from 15,000 cells/cm^2^ to 30,000 cells/cm^2^ (rabbit: 15,000 cells/cm^2^; mouse & rat: 30,000 cells/cm^2^; all other genera: 20,000 cells/cm^2^), into poly-L-lysine coated dishes (5 μg/cm^2^) and fed with serum-free DMEM/ F12 culture medium supplemented with insulin, transferrin, selenium (ITS-X, 1% Life Technologies), Epidermal Growth Factor (EGF, 50 ng/mL; R&D Systems) and Fibroblast Growth Factor 2 (FGF2, 50 ng/mL; R&D Systems). This culture medium was changed every two days. After 10 days of treatment, spheres were harvested by gently shaking the medium and pooled in one culture well (2 cm^2^) for imaging.

### Immunocytochemistry

Immunocytochemistry was carried out to assess expression of nestin protein, known to be strongly expressed in OE-MSCs [14, 34] and neural proteins GFAP and MAP2. OE-MSCs (passage 6) were plated on glass coverslips at a density of 15,000 cells per cm^2^ in growth medium for approximately 48 h. The cells were then fixed in a paraformaldehyde solution (4%, Antigenfix, MM France) for 15 min and incubated for 1 h at RT with blocking solution (3% bovine serum albumin, 5% goat serum and 0.1% Triton X-100, Sigma Aldrich) in phosphate-buffered saline (PBS) solution. Glass coverslips were then incubated for 90 min at RT with the appropriate primary antibody diluted in the blocking solution (Table 2). For nestin detection, mouse monoclonal anti-nestin (Abcys) was used for rodent and rabbit polyclonal anti-nestin (Abcam) for all other studied genera. The cells were then rinsed 3 times in PBS and incubated for 60 min with the appropriate polyclonal secondary antibody (1:500, Jackson ImmunoResearch, Table 2). After several washes in PBS, cells were counterstained with 0.5 μg/mL Hoechst blue (33,258, Sigma-Aldrich) for 10 min and mounted with anti-fading medium (ProLong Diamond; Life Technologies). Negative control conditions were carried out by omitting the primary antibody. Pictures were acquired with an inverted microscope (Axio Imager, Carl Zeiss microscopy, Germany) and negative controls were used to adjust image acquisition parameters.

**Table 2:** Antibodies used for immunochemistry & flow cytometry

Target	Host	Supplier	Reference	Dilution	Secondary antibody
Nestin	Mouse	Abcys	VMA353	1:250	Alexa Fluor 488
Nestin	Rabbit	Abcam	ab7659	1:200	Alexa Fluor 488
Tenomodulin	Rabbit	Abcam	ab81328	1:500	Alexa Fluor 488
Scleraxis	Rabbit	Abcam	ab58655	1:500	Alexa Fluor 488
GFAP	Chicken	Abcam	ab4674	1:500	Alexa Fluor 488
MAP2	Chicken	Abcam	ab5392	1:500	Alexa Fluor 488
CD34	Rabbit	Abcam	ab150060	1:50	Alexa Fluor 488
CD44	Rabbit	Abcam	ab157107	1:50	Alexa Fluor 488
CD73	Rabbit	Abcam	ab175396	1:110	Alexa Fluor 488
Rabbit IgG	Rabbit	Abcam	ab171870	1:50	Alexa Fluor 488

*GFAP* Glial Fibrillary Acidic Protein, *MAP2* Microtubule-associated protein 2

### Flow cytometry analysis

Using flow cytometry, we analyzed expression of 3 surface markers. Two of them (CD44 and CD73) are known to be strongly expressed in human OE-MSCs [14] while the third one, CD34, is not expressed. Cells were washed twice in PBS and then harvested using TrypLE™ Select Enzyme (Life Technologies). Then, the cells were centrifuged (300 x g, 5 min), resuspended in cold blocking solution (10% FBS in PBS) and centrifuged again. Cells were paraformaldehyde-fixed for 15 min RT (4%, Antigenfix), washed twice in blocking solution and permeabilized in cold methanol (90%, − 20 °C) 30 min at 4 °C, before being washed twice in blocking solution. Cells were then incubated 20 min RT with primary antibodies against CD34, CD44 or CD73 (Table 2) diluted in blocking solution or incubated with the corresponding isotype control (rabbit IgG, Table 2) at the same concentration, as a negative control. Cells were then washed 3 times by centrifugation (600 x g, 5 min) and incubated 20 min RT in the absence of light with the corresponding secondary antibody diluted in blocking solution (Table 2). After three washes, cells were immediately processed for flow cytometric analysis. Acquisitions were performed on a FACSCanto II flow cytometer (BD Biosciences) using BDFACSDiva software. At least 10,000 events were recorded for each analysis and measures were performed in duplicate. Percentages are presented after the subtraction of isotype background and refer to the total living population analyzed.

### Clonal efficiency assay

The assay was carried out by plating OE-MSCs (passage 7) from one representative culture per genus in 6-well plates at a density ranging from 10 to 320 cells/well in triplicate by using a 1:2 serial dilution in growth medium. After plating, the dishes were placed in an incubator (37 °C, 5% CO2) and left untouched for 7 days before being paraformaldehyde-fixed (4%, Antigenfix) during 15 min at room temperature (RT). Colonies were stained for 15 min using crystal violet and then manually counted. For each sample, clonal efficiency (% of clonogenicity) was calculated as follows: (mean number of colonies/total number of seeded cells) × 100. When too many colonies overlapped, counting was not performed.

### In vitro proliferation assay

The assay was performed on OE-MSCs for each studied genus, 2 months (10 passages) and 3 months (20 passages) after the initial plating. Cells from one representative culture per genus were seeded at a density of 200 cells/cm^2^ in 24-well plates in triplicate in growth medium, during 8, 24, 48, 72 or 96 h. After being paraformaldehyde-fixed (4%, Antigenfix) for 15 min at RT and stained with Hoechst blue (0.5 μg/ml, Sigma-Aldrich), the cells were counted for each of the 6 tested conditions, using an inverted microscope (Zeiss microscopy) and a computer procedure (ImageJ). The population doubling-time was calculated using “Doubling-Time.com” (Roth V. 2006).

### In vitro mesodermal differentiation assays

Human OE-MSCs have been previously described to be able to differentiate in vitro into different types of mesodermal cells (Murrel et al., 2005; Delorme et al., 2010). These characteristics in OE-MSCs from rat, rabbit, dog and horse were assessed. For osteogenic and chondrogenic differentiation, olfactory stem cells were grown using same techniques as previously described [14]. To evaluate osteogenic differentiation, cells cultures were fixed in a paraformaldehyde solution (4%) for 15 min and stained with von Kossa (Bio-Optica) or Alizarin Red stain (ScienCell), according to manufacturers’ instructions. For chondrogenic differentiation, cells were grown in pellets and fixed in 10% buffered formalin (pH 7.4), routinely processed and paraffin-embedded. Four μm thick sections were cut and stained with Toluidine blue (Diapath) or Alcian blue (Bio-Optica) according to instructions.

For tenogenic differentiation, we adapted different protocols used for MSC differentiation. Thus, 30.000 OE-MSCs were grown on a 5 μg/cm^2^ collagen-I matrix (Sigma/Aldrich) in DMEM without FBS, 50 ng/ml GDF-5 (R&D Systems), 50 ng/ml GDF-7 (R&D Systems) and 20 ng/ml TGF-B3 (R&D Systems) for 7 days [36–38]. To evaluate tenogenic differentiation, cells were fixed as previously described and immunochemistry against Tenomodulin and Scleraxis proteins carried out using the same procedure described above, with the appropriate antibodies (see Table 2).

## Results

### Biopsies of olfactory mucosa on living animals

Olfactory mucosa biopsies were successfully performed on living animals from all genera except mice, with some differences in collection and culture techniques (Table 1). Biopsied animals under anesthesia could recover within a few hours after surgery without any sign of pain or unwanted side effects. The only undesirable effect observed in all studied genera was nasal bleeding immediately following the biopsies, which was rapidly suppressed by applying a sterile gauze in the nose or on the bone window.

### Isolation and amplification of cells with fibroblastic morphology from olfactory mucosa biopsies

**Fig. 1:**
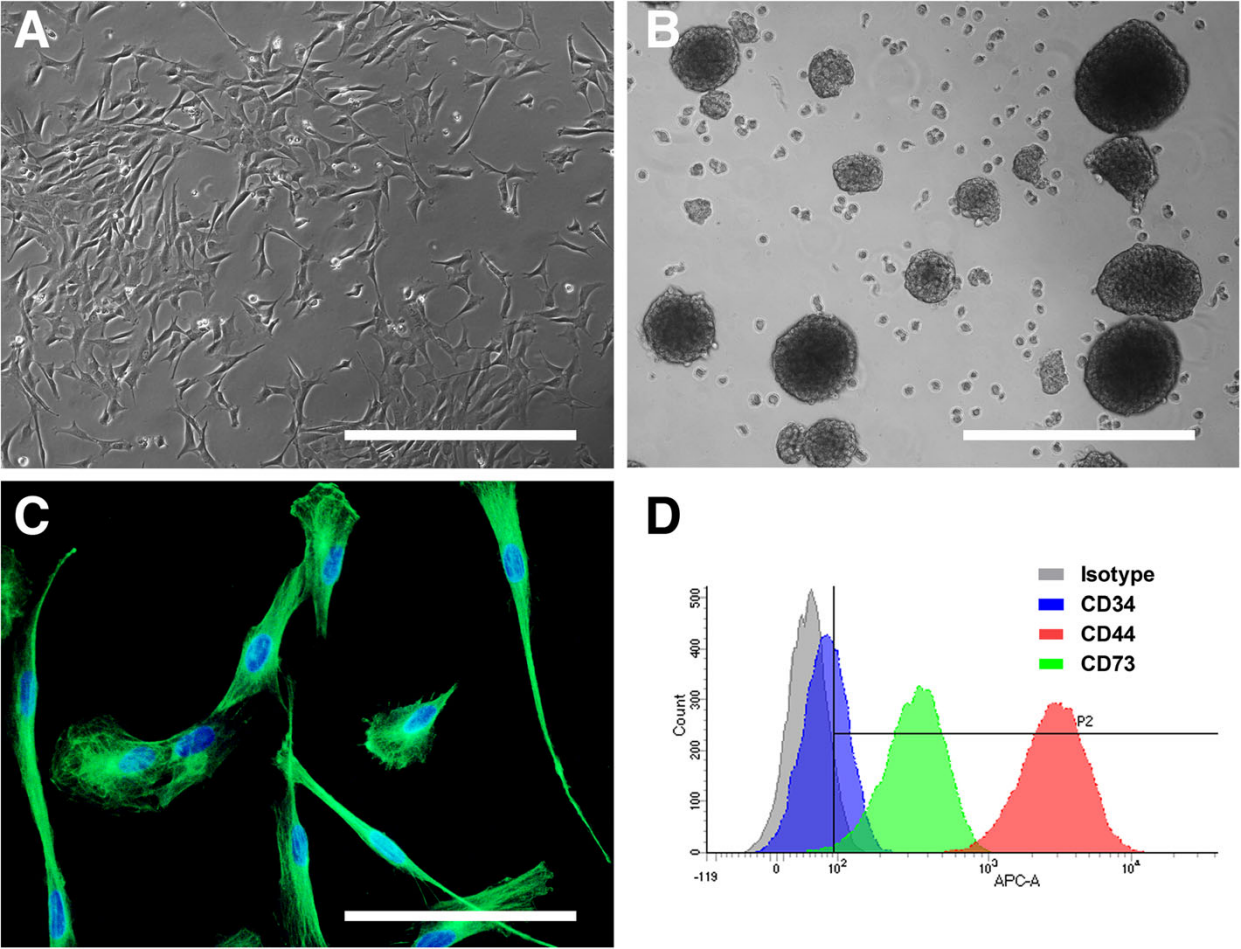
Morphology and stemness features of OE-MSCs from different mammalian genera. After 4 weeks in growth culture medium, olfactory mucosa explants formed homogeneous populations of adherent and highly proliferative cells exhibiting a mesenchymal-like fibroblastic morphology: examples of sheep (**a**). When grown under appropriate culture conditions, OE-MSCs could generate spheres: examples of rabbit (**b**). After seven passages, cells express the nestin protein (in green, (**c**) example of rabbit), a prominent marker of immaturity. OE-MSCs were immunostained with 3 surface markers, quantified using a flow cytometer and expression level compared to isotype: example of macaque (**d**). Each image is representative of multiple independent cultures of each species. Scale bar: 200 μm (**a** & **b**), 100 μm (**c**)

One to two weeks after biopsy, adherent cells with heterogeneous morphologies from each genus began to grow out of the explants and invaded the culture dish. After 2 passages, cultures became more homogenous and cells exhibited a fibroblastic morphology (Fig. 1a). Cells from mouse showed morphological changes with increasing passages or dilution associated with a difficulty in amplifying them. A vulnerability to dilution was also observed for all genera during the first steps of amplification, with a decrease in cell proliferation if split ratio exceeded 1:2 (data not shown).

### OE-MSCs from different mammalian genera display features of stemness

After amplification, two stemness and immaturity features that were previously described in human OE-MSCs were assessed: the ability to give rise to spheres and the expression of nestin. When grown under specific appropriate culture conditions, OE-MSCs from all genera could generate spheres (Fig. 1b).

For each genus, the entire population of OE-MSCs expressed the nestin protein, a prominent marker of immaturity, with similar intensities of expression across all genera (Fig. 1c).

Finally, OE-MSCs were successfully transfected to express GFP, a prerequisite for transplantation studies involving cell tracking (data not shown).

### OE-MSCs from different mammalian genera display similar expression of mesenchymal stem cell surface markers

We analyzed expression of 3 surface markers by flow cytometry. In each genus studied, CD34 expression was extremely low or undetectable with a percentage of cells expressing this marker inferior to 10 (Fig. 1d and Table 3). CD44 was highly and homogeneously expressed in OE-MSCs from all genera with a percentage of cells expressing this marker superior to 90 except in horse (68%). Expression of CD73 marker was more variable in the different genera. While percentage of cells expressing this marker is superior to 50% in cell population from rat, sheep, dog, gray mouse lemur and macaque, CD73 expression was low in OE-MSCs from rabbit (22%) and extremely low in horse cells (5.5%).

**Table 3:** Analysis of surface markers expression by flow cytometry

	% cells expressing markers (mean ± SEM)		
Genus	CD34	CD44	CD73
Rat	4.7 ± 0.6	98.6 ± 0.0	56.7 ± 11.0
Rabbit	0.7 ± 0.4	91.2 ± 1.0	22.0 ± 1.6
Sheep	5.0 ± 0.3	98.4 ± 0.0	64.3 ± 0.1
Dog	9.2 ± 2.2	96.1 ± 0.0	58.7 ± 15.2
Horse	2.4 ± 1.1	68.6 ± 5.8	5.5 ± 3.1
Gray mouse lemur	−1.0 ± 1.1	95.4 ± 0.0	93.6 ± 0.0
Macaque	1.7 ± 1.2	95.6 ± 1.0	73.9 ± 4.4

### High clonal efficiency and proliferation rate of OE-MSCs

**Fig. 2:**
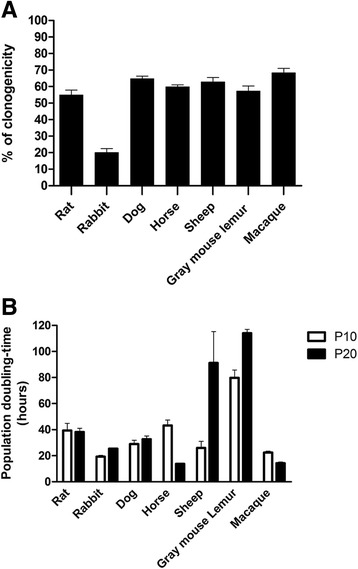
Assessment of proliferative and clonogenic properties of OE-MSCs from different mammalian genera. For each mammalian genus, a clonogenicity efficiency assay was carried out by plating OE-MSCs (passage 7) at low densities and by measuring the number of newly formed colonies after 7 days in culture. All tested genera displayed a high percentage of OE-MSCs capable of forming new colonies (% of clonogenicity, **a**). The population doubling-time (in hours) was measured for each mammalian genus after 2 months (10 passages) and 3 months (20 passaging) in culture (**b**). Most of OE-MSC populations display a high proliferation rate but genus specificities are observed. Values reported are the mean (+/− SEM) of three independent experiments carried out in triplicate, on one representative member of each genus

Evaluation of clonal efficiency after seeding of the cells at low density indicated that a high percentage of OE-MSCs from all studied genera could give rise to colonies after 7 days in culture, except mice OE-MSCs which were unable to generate new clonal populations (Fig. 2a). The percentage of OE-MSCs generating new colonies after seven passages ranged between 50% and 70% in rat, dog, horse, sheep, gray mouse lemur and macaque. Rabbit cells displayed a lower clonal efficiency (20%) in comparison to their outstanding proliferation rate (Fig. 2b). Assessment of the population doubling-time in short-term (passage 10, 2 months of culture) and long-term (passage 20, 3 months of culture) passages revealed that most OE-MSCs are quickly dividing cells. As shown in Fig. 2b, cells from all genera, except those derived from gray mouse lemur olfactory mucosa, displayed a doubling-time ranging from 20 and 40 h after 10 passages. Except for the sheep and gray mouse lemur, the doubling-time remained stable after 20 passages and even decreased in horse and macaque cells.

### In vitro assessment of differentiation abilities of OE-MSCs

**Fig. 3:**
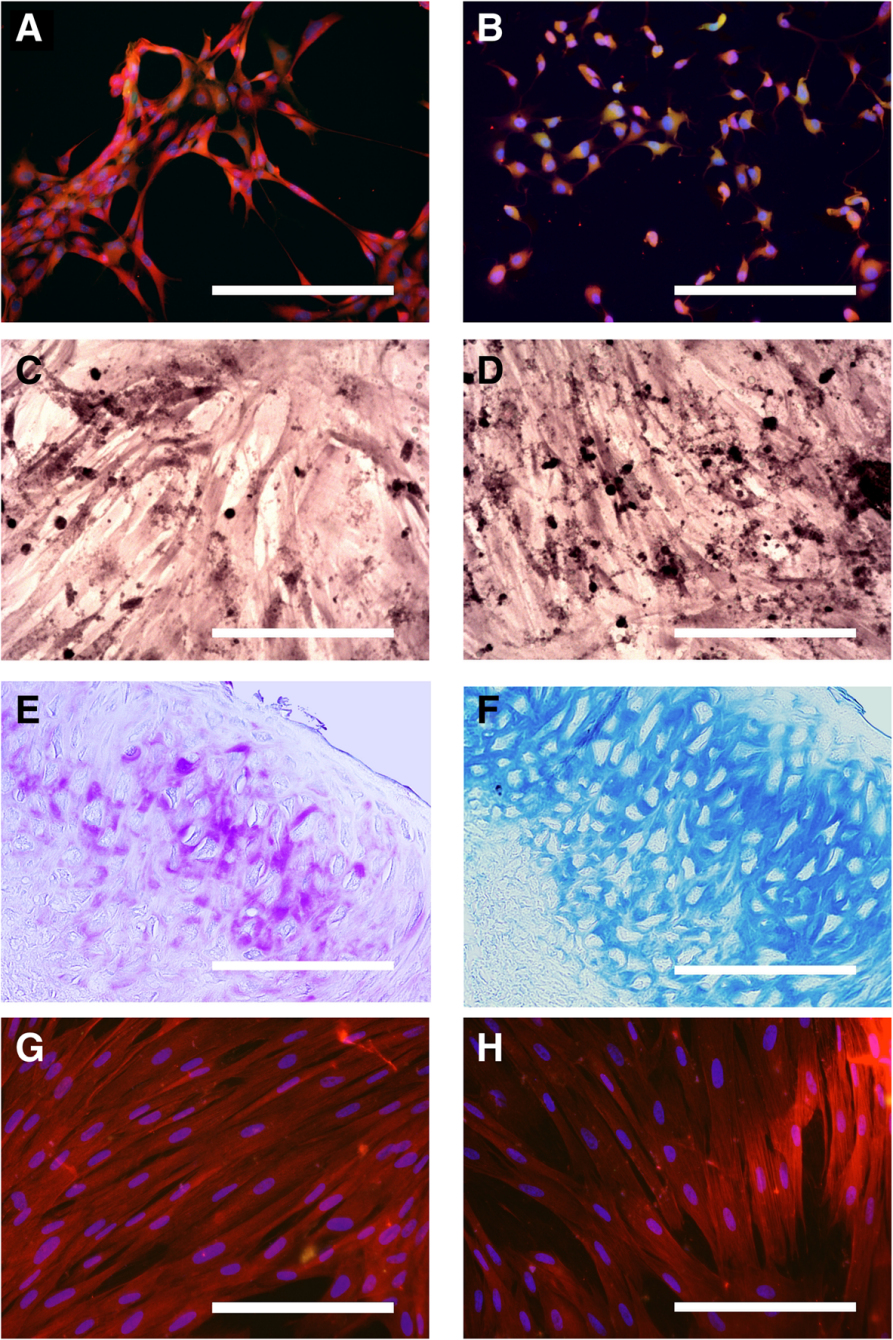
Assessment of neural and mesodermal differentiation abilities of OE-MSCs in vitro**.** Multipotency was assessed in OE-MSCs from rat, rabbit, dog and horse. Expression of the neural proteins GFAP (**a**) and MAP2 (**b**) in red in undifferentiated rat OE-MSCs. Bone differentiation was assessed using Red Alizarin (**c**) and Von kossa (**d**) stainings. Dog OE-MSCs were positively labeled in red (**c**) and in black (**d**) using these procedures. Chondrogenic differentiation was assessed using Toluidine Blue (**e**) and Alcian Blue (**f**) stainings. Horse OE-MSCs were positively labeled in purple (**e**) and in blue (**f**) using these procedures. Expression of the tenocytic markers Scleraxis protein (**g**) and Tenomodulin (**h**) in red in rabbit OE-MSCs. Each image is representative of multiple independent cultures of each species. Scale bar: 200 μm

Prior to any differentiation, we observed that cells from four studied genera (rat, rabbit, dog and horse) expressed neural proteins GFAP (Fig. 3a) and MAP2 (Fig. 3b) at a basal state. Subsequently, differentiation assays of these cells into cells of the mesodermal lineage were performed. We found they could be induced to express bio-chemical features specific to osteoblasts (Fig. 3c & d), chondroblasts (Fig. 3e & f) and tenoblasts (Fig. 3g & h). Detailed results of differentiation are reported in Table 4. Osteoblast differentiation was ineffective in rat OE-MSCs but cells from the other studied genera were reactive for Red Alizarin and Von kossa stainings (Fig. 3c & d). Only horse and rat OE-MSCs were reactive for both Toluidine Blue (E) and Alcian Blue (F) stainings after chondrogenic differentiation. Dog OE-MSCs were reactive only for Alcian Blue while cells from rabbit did not display any sign of differentiation. Tenogenic differentiation was positive for all the four genera studied and all the cells expressed both scleraxis protein and Tenomodulin.

**Table 4:** In vitro assessment of mesodermal differentiation of OE-MSCs

Tissue	Antibody / Staining	Rat	Rabbit	Dog	Horse
Bone	Von Kossa	–	–	+	+
	Alizarin Red	–	+	+	+
Cartilage	Alcian Blue	+	–	+	+
	Toluidine Blue	+	–	–	+
Tendon	Scleraxis	+	+	+	+
	Tenomodulin	+	+	+	+

## Discussion

The present study showed for the first time that OE-MSCs can be extracted from eight different mammalian genera and amplified to get tens million cells in few weeks. Cells displayed similar features to their human counterparts: a fibroblastic morphology, a robust expression of nestin, an ability to form spheres, a similar expression of surface markers (CD44, CD73), a high proliferation rate and an ability to be induced into cells of the mesodermal lineages; although some genus-specific properties were observed. These sampling and amplification techniques may permit autologous grafts for preclinical studies or clinical use in veterinary medicine in a wide variety of models.

Unlike previous methods used for the collection of rat olfactory mucosa, for the purposes of this study techniques were developed to harvest the appropriate tissue without sacrificing the donor animal [20] (Table 1). It is important to note that, except for occasional bleeding that can be prevented, no visible side effects were observed when a postoperative antibiotic treatment was applied. These results confirm those previously obtained in rats showing no disorder of the sense of smell after biopsy [34]. Among the eight tested genera, only the mouse olfactory mucosa was not collected on living animals. Surgery was considered too invasive for such a small nasal cavity and, accordingly, only syngeneic or allogeneic grafts can be considered for use in this genus.

### OE-MSCs: Similar characteristics shared by the different genera

Despite some differences between genera, the results indicated that the various mammalian OE-MSCs displayed similar characteristics to those previously described in their human counterparts [14]. Noticeably, these methods were successful in obtaining many highly proliferative cells. Interestingly, though it is not possible to ascertain the absolute purity of stem cells and exclude potential contamination with other cell types, such as the olfactory ensheathing cells, the populations of OE-MSCs were highly homogeneous, when comparing cell morphology, expression of nestin and surface markers CD34 and CD44 according to previous studies in human and rat [14, 20, 34]. While the expression of CD34 and CD44 in cells from all genera was similar with the data from human OE-MSCs, we observed higher variability for CD73. First, this marker could not be detected at significant levels in horse OE-MSCs. Although we could not exclude that the antibody used was not able to recognize the horse protein, this observation agrees with other research groups who could not detect CD73, using multiples antibodies, in different horse MSC subtypes that normally express this marker in humans and rodents [39–41]. While the same technical issues may happen with cells from rabbit, CD73 is not, weakly or highly expressed in rabbit MSCs according to three different studies [42–44]. However, our results confirm the data of another research team which observed a similar percentage of positive cells (28%) [45]. In addition to the expression of these markers, the ability to generate spheres, the strong mitotic activity that remained high in most cases after 20 passages, and the high clonal efficiency rate, serve as indisputable proofs of the stemness features of these cells.

For unknown reasons, mouse olfactory stem cells were the most difficult to grow and amplify and were not tested for following evaluations. Accordingly, in addition to the complexity of collecting olfactory mucosa biopsies in living mice, autologous transplantations appear highly difficult in this genus.

### *OE-MSCs:* Interesting in vitro characteristics for in vivo applications

Compared to human OE-MSCs [14], cells from different genera displayed similar clonal ability (with the exception of rabbit cells), and shorter doubling-times, probably due to the study’s improved culturing techniques. Moreover, this study opens perspectives for future researches by demonstrating that these cells can be easily transfected to express GFP (data not shown). Thus, stem cells can be tracked in vivo after grafting to observe their differentiation, integration and interaction with the surrounding environment.

These perspectives are enhanced by the multipotency of OE-MSCs, an ability we evaluated in four species relevant for basic research or clinical veterinary practice While stem cells from rat, rabbit, dog and horse express at basal states two markers of neural cells, GFAP and MAP2, certainly due to their origin from an ectodermic tissue [21, 22], they could also differentiate into cells of mesodermal lineage. Overall, we confirmed the results of other studies evaluating differentiation abilities of OE-MSCs [13, 14, 46, 47].

Cells from these four genera could be induced in tenoblasts-like cells but the expression of bio-chemical features specific to osteoblasts and chondroblasts depend on genera. This variability may be potentially explained by inter-species sequence differences of differentiating factors and their receptors that can induce a loss of differentiation efficiency for some studied genera.

### OE-MSCs: An interesting tool for veterinary medicine

Among all stem cell candidates for regenerative therapies, those originating from bone marrow or adipose tissue are the most extensively studied [48, 49]. A recent study demonstrated the advantages but also the limitations associated with these particular types of stem cells [2]. Although bone marrow is known for containing a high number of MSCs, the collection procedure is complex, painful and may induce a non-negligible risk of infection or hemorrhage [50, 51]. Comparatively, collection of adipose tissue is less invasive and provides MSCs displaying similar properties [3, 52]. However, the quality of fat-derived stem cells seems subject specific [53–55], which limit their interest for cellular therapies.

The OE-MSCs presented in the current study are abundant in the olfactory mucosa and their proliferative abilities allow for rapid propagation [24, 47].

The therapeutic potential of OE-MSCs has been positively evaluated in various rodent models of tissue damage without inducing tumors, supporting the need for further assessment in clinical applications [25, 28, 32, 56]. In fundamental research, stem cells are commonly used in mice, rats, rabbits, gray mouse lemurs and macaques. Several studies have reported various applications for injured [9] or degenerative central nervous systems [4], orthopedic problems [57], cartilage defects [58], tendon-to-bone healing [59], and skeletal [60] and cardiac [7] muscle engineering and OE-MSCs, thanks to their multipotency, may be promising to treat such defects.

Moreover, cell-based treatments in large animal models are emerging [1, 2, 12]. Due to their anatomical, physiological and genetic similarities to humans, domestic animals or non-human primate represent a step towards clinical applications. Gray mouse lemurs and dogs are now commonly recognized as reliable natural models of Alzheimer-like diseases [61–64]. Osteoarticular diseases also play a major role in veterinary medicine, especially in dogs and horses [65, 66]. Techniques presented here opens perspectives for future researches on these natural models.

While the present study focuses on OE-MSCs, it can be pointed out that the olfactory mucosa may also be used as a source of another cell type of great interest for regenerative medicine, namely the olfactory ensheathing cells [47, 67, 68]. Olfactory ensheathing cells are central glia sharing common properties with immature Schwann cells [69]. They have been shown to promote axonal regeneration in the CNS [68, 70] and reduce neuro-inflammation [71] after being transplanted alone or in combination with stem cells.

## Conclusion

The current study confirms that OE-MSCs can be easily harvested from olfactory mucosa of most mammalian genera for use in autologous transplantation without any damaging side effects. Reported techniques of biopsy and culture can be used to obtain millions of nasal olfactory stem cells in a short time. Their outstanding ability to proliferate and stemness characteristics, associated with abilities to generate cells from different lineages, make these multipotent stem cells suitable tools for regenerative medicine. This study paves the way for i) the development of fundamental research on a wide variety of models of tissue injuries and ii) clinical trials to evaluate the therapeutic benefit of OE-MSCs. For domestic and/or companion animals, the development of such therapies shows great promise as it will positively impact the lives of veterinary patients, while promoting human applications.

## Abbreviation

GFP: Green Fluorescent Protein
MSC: Mesenchymal stem cell
OE-MSC: Olfactory ecto-mesenchymal stem cell
